# Critical chemistry manufacturing and controls considerations for mRNA lipid nanoparticle translation

**DOI:** 10.1186/s11671-026-04864-4

**Published:** 2026-08-13

**Authors:** Lanbo Liu, Yan Wang, Hua Gao, Jinghui Ruan, Wenna Lu, Tianzhu Liu, Xun Li, Guo-Qiang Zhang, Yating Xiao

**Affiliations:** 1 School of Molecular Medicine, Hangzhou Institute for Advanced Study, UCAS, Hangzhou, 310024 People’s Republic of China; 2 State Key Laboratory of New Pharmaceutical Preparations and Excipients, Key Laboratory of Medicinal Chemistry and Molecular Diagnosis of Ministry of Education, College of Pharmaceutical Sciences, Hebei University, Baoding, 071002 People’s Republic of China; 3 College of Life and Environmental Sciences, Hangzhou Normal University, Hangzhou, 311121 People’s Republic of China; 4 Zhejiang Hisun Pharmaceutical Co., Ltd., Taizhou, 318000 Zhejiang China; 5 Hangzhou Institute of Medicine (HIM), Chinese Academy of Sciences, Hangzhou, 310022 Zhejiang People’s Republic of China

**Keywords:** mRNA therapeutics, Lipid nanoparticles, Ionizable lipids, Manufacturing and controls, Critical quality attributes, Critical process parameters

## Abstract

Messenger ribonucleic acid (mRNA) therapeutics have advanced rapidly, but their translation remains constrained by delivery performance, manufacturing robustness, and regulatory expectations. Lipid nanoparticles (LNPs) are the most clinically established non-viral delivery platform, yet their successful development depends on a well-defined chemistry, manufacturing, and controls (CMC) strategy that links lipid chemotype and process parameters to critical quality attributes (CQAs) and, ultimately, clinical performance. This review examines translation-critical decisions in mRNA-LNP development, beginning with a comparison of ionizable and permanently cationic lipid chemotypes in terms of efficacy–tolerability trade-offs, biodegradability, and immune activation. We then contrast LNPs with polymeric and hybrid carriers, with emphasis on characterization burden, scalability, and regulatory precedent. Manufacturing approaches, including microfluidic scale-out and impinging-jet scale-up, are further discussed in relation to their effects on CQAs, critical process parameter (CPP) sensitivity, comparability, and cost. Beyond formulation and processing, we highlight the need for orthogonal analytical characterization, physiological stability assessment in plasma or serum, and stability-indicating profiling of lipid impurities and mRNA–lipid adducts. We also discuss AI/ML-ready metadata standards and early comparability planning for scale-up and post-approval changes. Together, these considerations provide a practical CMC-oriented framework for aligning formulation design, manufacturing control, and clinical translation of mRNA-LNP products.

## Introduction

**Fig. 1:**
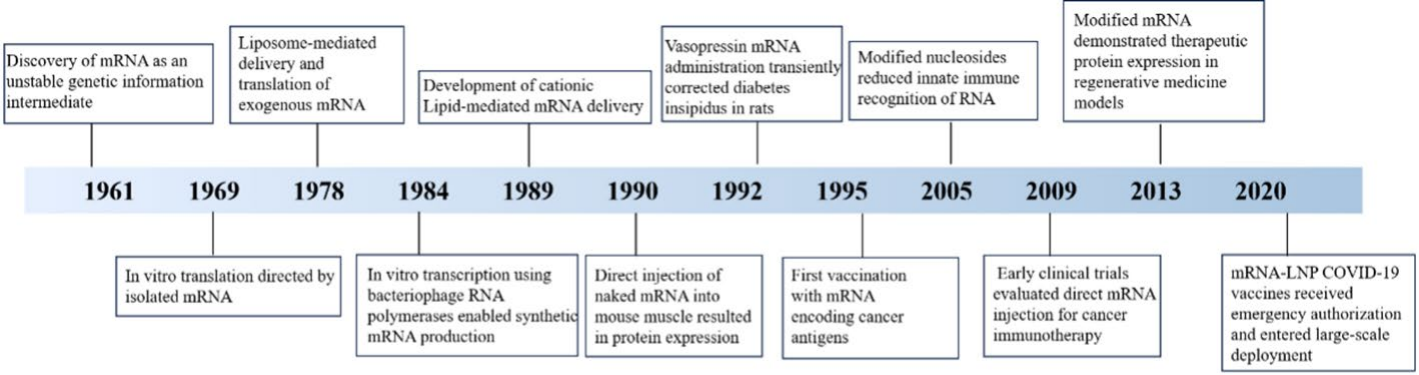
Key milestones in the industrial translation of mRNA therapeutics

mRNA is a single-stranded RNA template that carries genetic information from DNA to the cellular machinery responsible for protein synthesis. Its therapeutic value lies in the ability to deliver designed RNA sequences into cells to modulate or restore protein expression. This approach has opened new possibilities across a wide range of therapeutic areas, including infectious diseases, oncology, inherited disorders, and rare diseases. The concept of mRNA-mediated protein synthesis was established in the 1960s [1, 2]. Subsequent advances in nucleoside modification, cap analog design, and in vitro transcription technologies have substantially improved the stability and translational efficiency of synthetic mRNA (Fig. 1). This is reflected in at least seven marketed or authorized mRNA products since 2020 (Table 1). These breakthroughs have enabled the rapid development of mRNA-based vaccines and therapeutics [3].

**Table 1:** Currently marketed mRNA products worldwide

Brand	Company	Indication	Approval (yr)
COMIRNATY®	Pfizer-BioNTech	COVID-19	2020
Spikevax®	Moderna	COVID-19	2021
AWcorna (ARCoV)	Walvax / Abogen / Academy of Military Medical Sciences	COVID-19	2022
DAICHIRONA®	Daiichi Sankyo	COVID-19 booster	2023
KOSTAIVE®	Arcturus / Meiji Seika (Japan); CSL Seqirus	COVID-19 (primary and booster, per label)	2023
SYS6006	CSPC Pharmaceutical	COVID-19 (booster)	2023
mRESVIA®	Moderna	Respiratory syncytial virus (RSV) prevention in adults	2024

The COVID-19 pandemic highlighted the transformative potential of mRNA platforms, with mRNA vaccines gaining emergency authorization and large-scale deployment within unprecedented timelines. Beyond vaccines, mRNA is being actively explored for protein replacement therapies and genome editing, supported by innovations in RNA nanotechnology and delivery science. Compared with DNA-based approaches, mRNA offers several advantages. It acts directly in the cytoplasm without requiring nuclear entry, avoids the risk of genomic integration, and enables transient but controllable protein expression [4]. Furthermore, the production of mRNA can be standardized and scaled efficiently, making it suitable for rapid response to emerging medical needs.

Despite these advantages, the translation of mRNA medicines into clinical and commercial products faces critical challenges. Naked mRNA is inherently unstable, prone to rapid degradation by nucleases [5], and can trigger innate immune responses via Toll-like receptors [6]. Its large size and strong negative charge hinder efficient cellular uptake [7, 8]. These limitations underscore the central role of delivery systems. Lipid nanoparticles (LNPs) have emerged as the most advanced and commercially validated vectors [9], while polymeric and hybrid systems remain under active investigation. Delivery systems must simultaneously enable cytosolic delivery and satisfy industrial constraints, including reproducible large-scale manufacturing, quality control, supply chain robustness, and regulatory compliance [10]. In this review, we therefore focus on translation-critical design and CMC decisions rather than providing an encyclopedic overview of all carrier classes.

From an industrial perspective, mRNA delivery research has shifted from proof-of-concept studies to questions of manufacturability, supply chain robustness, and regulatory acceptance. Achieving consistent particle size distribution, encapsulation efficiency, physiological stability, stability-indicating impurity control, and long-term storage stability is essential for regulatory approval and global distribution [11]. Therefore, the integration of mRNA technology with scalable delivery platforms-particularly LNPs [12]-represents a critical step in moving RNA therapeutics from laboratory innovation to commercial reality.

Recent reviews have summarized LNP design principles and biological performance. Here we focus on the practical decisions that often determine whether an mRNA delivery concept can be scaled and filed: how lipid chemotype choice (ionizable vs permanently cationic) maps to efficacy-tolerability-clearance trade-offs; how manufacturing routes (microfluidic scale-out, impinging-jet scale-up, and bulk mixing) influence CQAs and CPP sensitivity; and how early CMC planning (raw-material qualification, orthogonal analytics, physiological-stability testing, control strategy, and comparability) supports scale-up, site transfer, and post-approval changes. We also provide a focused comparison of LNPs and polymeric/hybrid carriers to clarify where non-lipid systems are promising and where translation is commonly constrained. For clarity, we use industrial feasibility to mean reproducible GMP manufacture at the intended scale within predefined CQA specifications, and commercial viability to mean industrial feasibility plus acceptable cost of goods, supply resilience, and a practical stability/distribution profile.

## Nanoliposomes for RNA delivery

### Lipid vectors for mRNA delivery: historical overview and current challenges

Efficient delivery of messenger RNA (mRNA) remains one of the main barriers to clinical translation. Early doubts about mRNA drugs arose from its instability and poor in vivo performance [13]. Since the 1970s, however, lipid-based nanoparticles (LNPs) have gradually become the leading solution, supported by advances in lipid chemistry and formulation technologies [14].

The physicochemical features of mRNA—its large size (1–15 kb) and strong negative charge—make direct cellular uptake inefficient. Inspired by natural membrane lipids, synthetic molecules such as cationic lipid N-[1-(2, 3-dioleyloxy)propyl]-N, N,N-trimethylammonium chloride (DOTMA) [15], 1,2-dioleoyl-3-trimethylammonium-propane (DOTAP) [16], and newer degradable lipidoids [17, 18] have been developed to improve encapsulation and intracellular delivery. These systems now form the basis of clinical mRNA formulations. From an industrial standpoint, remaining challenges include reproducible large-scale production, long-term stability, and compliance with evolving regulatory standards.

### Functional classes of lipid vectors for mRNA delivery

#### Cationic lipids

First-generation cationic lipids, such as DOTMA and DOTAP, enabled RNA complexation through permanently charged quaternary ammonium headgroups [19, 20]. Their strong electrostatic interaction with nucleic acids and ability to form lipoplexes provided important early proof of concept for nucleic acid delivery. However, their constitutive positive charge can promote nonspecific serum interactions, complement activation, and membrane perturbation, which limits systemic tolerability and has restricted broader clinical use [21].

#### Ionizable lipids

Ionizable lipids now form the core of most LNP platforms. At physiological pH, they are largely neutral, but they become protonated under acidic conditions. This pH-responsive behavior enables efficient RNA encapsulation during low-pH mixing, reduces nonspecific interactions in circulation, and promotes endosomal escape after cellular uptake. Clinically used examples include ALC-0315 in COMIRNATY® and SM-102 in Spikevax®, while widely studied research scaffolds include DLin-MC3-DMA, DLin-DMA, and DLin-KC2-DMA. More recent lipid designs incorporate degradable linkers to reduce tissue retention and use formulation rules or compositional codes to guide organ tropism. The efficacy–tolerability trade-offs and translational implications of ionizable and permanently cationic lipids are compared in Table 2. These advances underpin marketed mRNA vaccines and are being extended to in vivo gene editing and extrahepatic delivery.

**Table 2:** Benchmarking ionizable versus permanently cationic lipids for mRNA delivery (efficacy-safety-translation trade-offs)

Dimension	Ionizable lipids	Permanently cationic lipids	Translational implication/ mitigation
Net charge at pH 7.4	Near-neutral	Positively charged	Ionizable lipids reduce nonspecific protein binding; cationic lipids increase serum interactions
Encapsulation mechanism	Protonated at low pH during mixing	Electrostatic complexation at physiological pH	Ionizable systems often require tighter pH control during mixing
Systemic tolerability	Generally improved vs cationic (not risk- free)	More limited for systemic dosing	Preference for ionizable chemotypes in systemic products
Immune stimulation risk	Depends on headgroup/impurities/dose	Often higher via complement and membrane perturbation	Mitigate via degradability, impurity control, and dosing strategy
Biodegradability and clearance	Increasingly engineered (cleavable linkers)	Often less biodegradable (chemotype dependent)	Degradable designs reduce accumulation but add impurity-control and stability-validation requirements
Potency window	Often high with optimized pKa/tails/linkers	Can be potent in local/IM settings	Balance potency with tolerability margin
Clinical/industrial precedent	Strong (vaccines; siRNA LNP precedent)	Limited systemic precedent; niche use	Regulatory familiarity favors ionizable LNPs
Common attrition drivers	Reactogenicity, liver signals, stability, CMC reproducibility	Dose-limiting toxicity, complement activation	Attrition often reflects narrow therapeutic index + CMC sensitivity

#### Composition of LNP-mediated mRNA delivery systems

Typical mRNA-LNPs contain four components: the RNA payload, helper lipids, cholesterol, and PEGylated lipids [22]. Each component affects stability, circulation, cellular uptake, and ultimately in vivo performance [14]. PEG-lipid anchor length controls de-PEGging kinetics and therefore influences cellular uptake and biodistribution (Table 3). Because modest shifts in composition and processing can propagate into critical quality attributes (CQAs)-such as particle size distribution, encapsulation efficiency, potency, impurity profile, and stability-industrial scale-up requires tight control of lipid quality and process windows under a Quality-by-Design (QbD) framework.

**Table 3:** Clinically validated and translation-relevant lipid components for mRNA-LNPs

Component class	Representative examples	Translational maturity	Primary role in LNP
Ionizable lipid (core)	ALC-0315; SM-102; Dlin-MC3-DMA	Marketed/clinically validated	Encapsulation at low pH; near- neutral circulation; endosomal escape
Helper phospholipid	DSPC; DOPE (context- dependent)	Clinically established excipients	Structural stability (DSPC); fusogenicity /escape (DOPE)
Sterol	Cholesterol	Clinically established	Membrane packing, stability, permeability tuning
PEG-lipid	DMG-PEG2000; DSPE-PEG2000; ALC-0159 family	Clinically established	Stealth during circulation; controls size; de-PEGging kinetics
Emerging motifs	Biodegradable linkers; SORT lipids; targeting ligands	Emerging/early clinical or preclinical	Clearance, extrahepatic targeting, immune tuning

#### Functional lipid design

LNP performance can be rationalized by three coupled modules: headgroups set ionization behavior and RNA affinity (pKa window in the mid-6.2–6.8 is often effective), hydrophobic tails determine membrane curvature and phase propensity, and linkers govern stability and biodegradability [23, 24]. Industrial development adds further requirements, including GMP-grade lipid synthesis, impurity control, batch-to-batch consistency, and release testing aligned with QbD principles. Stimuli-responsive lipids, such as pH- or redox-cleavable variants, and ligand-modified systems broaden the formulation design space, but they must still demonstrate sufficient manufacturing robustness and shelf-life stability.

#### Clinical translation and industrial outlook

Recent progress has moved lipid technologies into the clinic. DOTAP-based LNPs have reached early-phase cancer trials [25], while ionizable lipids underpin in vivo CRISPR therapies such as NTLA-2001 [26]. Responsive designs—such as pH- or redox-sensitive lipids [27–30] and organ-targeting (SORT) strategies [31–33]—are expanding the range of applications.

However, several key issues remain to be addressed, including hepatotoxicity, immune stimulation, and high manufacturing costs [22, 34]. Industrial efforts now focus on biodegradable lipid chemistries, controlled rapid-mixing processes, and improved formulation stability to reduce cold-chain requirements. These advances are essential to make mRNA delivery platforms commercially viable.

#### Polymeric and hybrid delivery systems for mRNA: translational status and CMC hurdles

Although LNPs currently dominate clinical translation, polymeric and hybrid carriers continue to be explored for applications in which LNPs may be less suitable, such as selected extrahepatic targets, repeated dosing, or alternative routes of administration. Polymeric systems, including polyplexes, biodegradable polycations, dendrimer-like architectures, and polymer–lipid hybrids, offer flexible chemistry and may improve physical stability in certain formulations. In practice, however, their translation is often constrained not only by biological potency, but also by CMC complexity.

From a CMC perspective, polymeric carriers can introduce additional heterogeneity, including molecular-weight distributions, architectural variability, and residual catalysts or monomers. These factors complicate specification setting, impurity control, and comparability assessment. By contrast, LNPs, although still complex, benefit from a more established characterization toolkit and clearer regulatory precedent from lipid-based nanomedicines. For many polymeric systems, batch-to-batch consistency depends on tight control of polymer synthesis and purification. Extensive impurity profiling and orthogonal analytical methods are also needed to link material attributes with CQAs and functional potency.

Accordingly, polymeric/hybrid carriers will likely expand the delivery toolbox, but translation benefits from an early, realistic plan: a clear clinical rationale for moving beyond LNP platforms, a scalable and GMP-ready synthesis/purification route with defined impurity controls, and a comparability strategy that connects material attributes to nanoparticle CQAs and potency. A summary of these trade-offs is provided in Table 4.

**Table 4:** LNPs versus polymeric/hybrid carriers: translational bottlenecks and regulatory acceptance

Dimension	LNPs	Polymeric/hybrid carriers	Translation implication
Clinical precedent	Strong (vaccines; siRNA LNP precedent)	More limited for systemic mRNA delivery	Precedent reduces regulatory uncertainty for LNPs
CMC characterization burden	High but increasingly standardized (size, EE, potency, composition)	Often higher heterogeneity (MW distribution, architecture)	Heterogeneity complicates specifications and comparability
Scale-up	Mature toolset (microfluidics, TFF, sterile filtration)	Depends on polymer synthesis + assembly route	Polymer supply and impurity control can dominate timelines
Raw material qualification	Lipid impurities/oxidation/PEG-lipid variability	Residual monomers, catalysts, polydispersity	Qualification strategy differs; both can be challenging
Stability	Cold-chain often required; lyophilization emerging	May be improved in some formats; product- specific	Stability claims must be evidence- based and product- specific
Key risks	Immune stimulation, liver signals, PEG immunogenicity	Toxicity of cationic polymers, complement activation	Risk mitigation can determine feasible dose
Regulatory acceptance	Growing guidance and precedents	Case-by-case; fewer precedents	Regulatory path may require more extensive justification

### mRNA delivery: overcoming the challenges

**Fig. 2:**
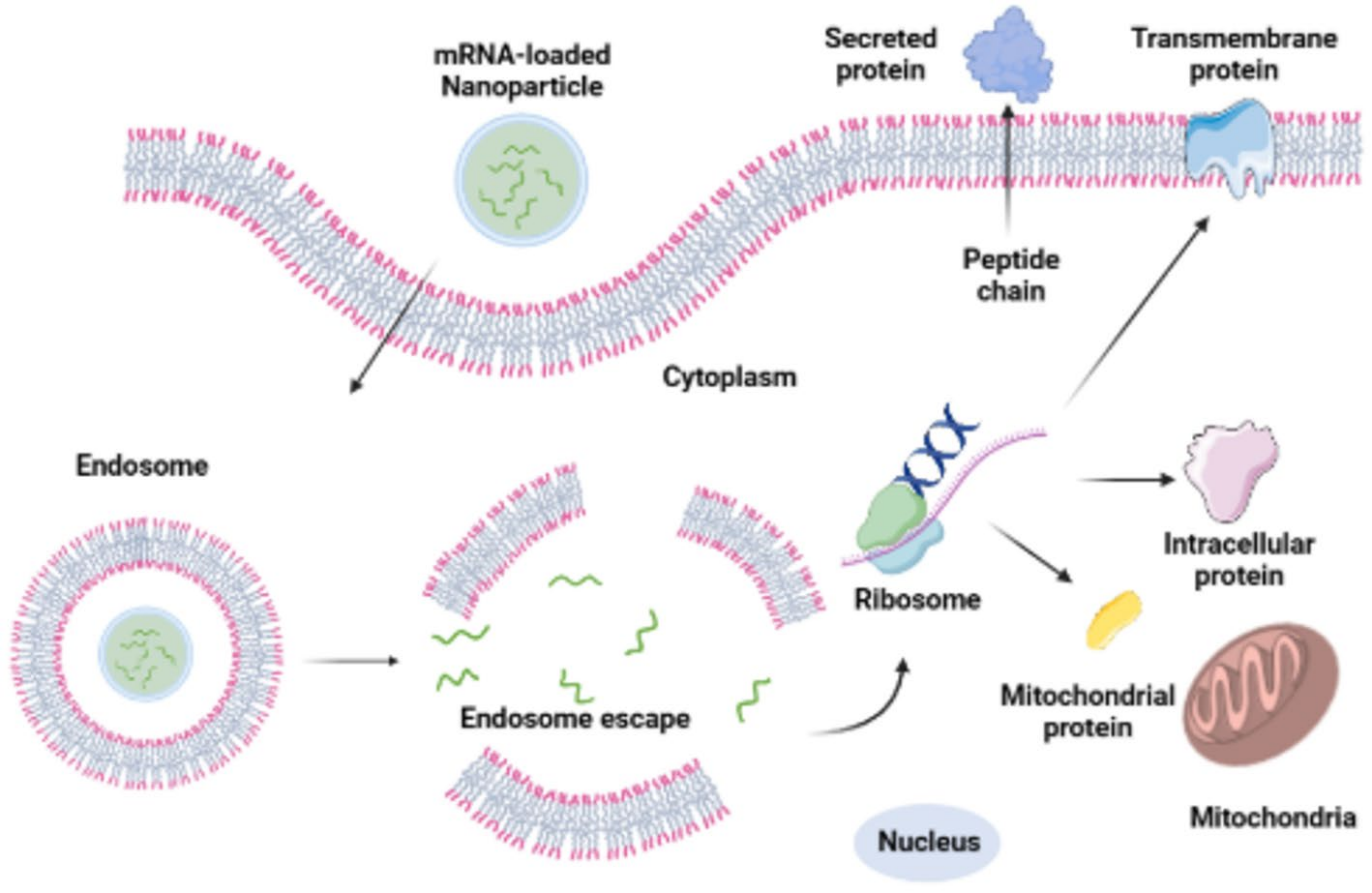
Schematic of cellular uptake of mRNA-LNPs and subsequent protein expression [4]. Copyright 2020, with permission from Elsevier

mRNA has become an important therapeutic modality, but its large molecular weight (300–5,000 kDa) and strong negative charge make delivery far more complex than for small oligonucleotides such as antisense or siRNA (4–15 kDa) [35, 36]. While certain immune cells, such as dendritic cells, can internalize mRNA [37, 38], most tissues require engineered carriers. For vaccines, protein replacement, or genome editing, broad and efficient delivery remains the central technical barrier [39, 40]. From a development perspective, LNPs have emerged as the most practical solution. By encapsulating mRNA, LNPs protect it from enzymatic degradation and promote cellular uptake through endocytosis. They also facilitate endosomal escape, allowing mRNA to be released into the cytosol for translation (Fig. 2). LNPs are modular systems that typically contain ionizable or cationic lipids, helper phospholipids, cholesterol, and PEG-lipids. This composition can be adjusted to tune stability and biodistribution. From an industrial perspective, the key challenge is to convert these laboratory formulations into reproducible, GMP-compliant products.

mRNA delivery remains challenging due to its size, polyanionic nature, and innate immune sensing. LNPs address these barriers by enabling high encapsulation, cellular uptake, and cytosolic release; however, translation requires balancing potency with tolerability and manufacturability, and establishing a CMC control strategy that maintains CQAs across scale and site changes.

**Fig. 3:**
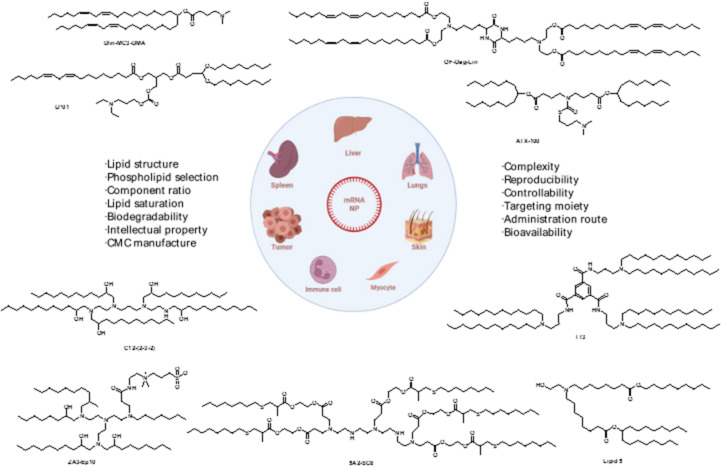
Representative lipid structures used for mRNA delivery. The central schematic summarizes reported cellular or organ tropism of selected delivery materials, while the surrounding labels highlight key design considerations for new lipid development

The field of lipid-mediated nucleic acid delivery has benefited substantially from earlier work on siRNA and plasmid DNA vectors. Early generations of cationic lipids such as DOTMA and DOTAP demonstrated proof-of-concept feasibility [41–44], but their limited safety profiles restricted broader application. Subsequent progress led to the development of ionizable lipids such as DLin-MC3-DMA [45], which was employed in the first FDA-approved siRNA drug, ONPATTRO®. Newer candidates including C12-200 [46] and cKK-E12 [47] further improved tolerability and enabled more precise organ targeting (Fig. 3). From an industrial standpoint, two strategies have become predominant: one involves adapting existing siRNA lipid systems for mRNA delivery [48, 49], while the other focuses on systematically optimizing lipid composition, including the ratios of ionizable lipids, PEG-lipids, cholesterol, and helper phospholipids, to maximize encapsulation efficiency and protein expression [50, 51].

The approval of ONPATTRO® provided regulatory validation for lipid-based nanoparticle systems in nucleic acid therapeutics [52–54]. The rapid deployment of mRNA vaccines against COVID-19 further showed that large-scale manufacturing and global distribution of LNP products are achievable when supported by robust process technologies, including microfluidic manufacturing [55–57]. Together, these milestones established both clinical feasibility and a development roadmap for future mRNA medicines.

Current product development efforts focus on balancing efficacy, safety, and manufacturability [58, 59]. Biodegradability is an important part of this balance. Lipids such as L319 and the ATX series have been designed with ester linkages in their hydrophobic chains to promote faster hepatic clearance and reduce long-term accumulation [60, 61]. Lipid tail architecture is equally important, since the degree of acyl-chain unsaturation governs fusogenicity, phase behavior, and biodistribution; incorporation of cis-unsaturated chains, exemplified by OF-02 derived from the cKK-E12 framework, can enhance in vivo protein expression [53, 62]. In addition, process control has emerged as a critical determinant of industrial translation, with microfluidic and continuous manufacturing methods enabling consistent particle size, high encapsulation efficiency, and improved batch-to-batch reproducibility [56, 63, 64]. Finally, product stability continues to present a challenge, particularly due to reliance on stringent cold-chain logistics. Lyophilization strategies and optimization of PEG-lipid components are therefore being actively explored to improve storage stability and facilitate broader clinical access.

## Advances in mRNA therapeutics

### Protein replacement therapy

**Fig. 4:**
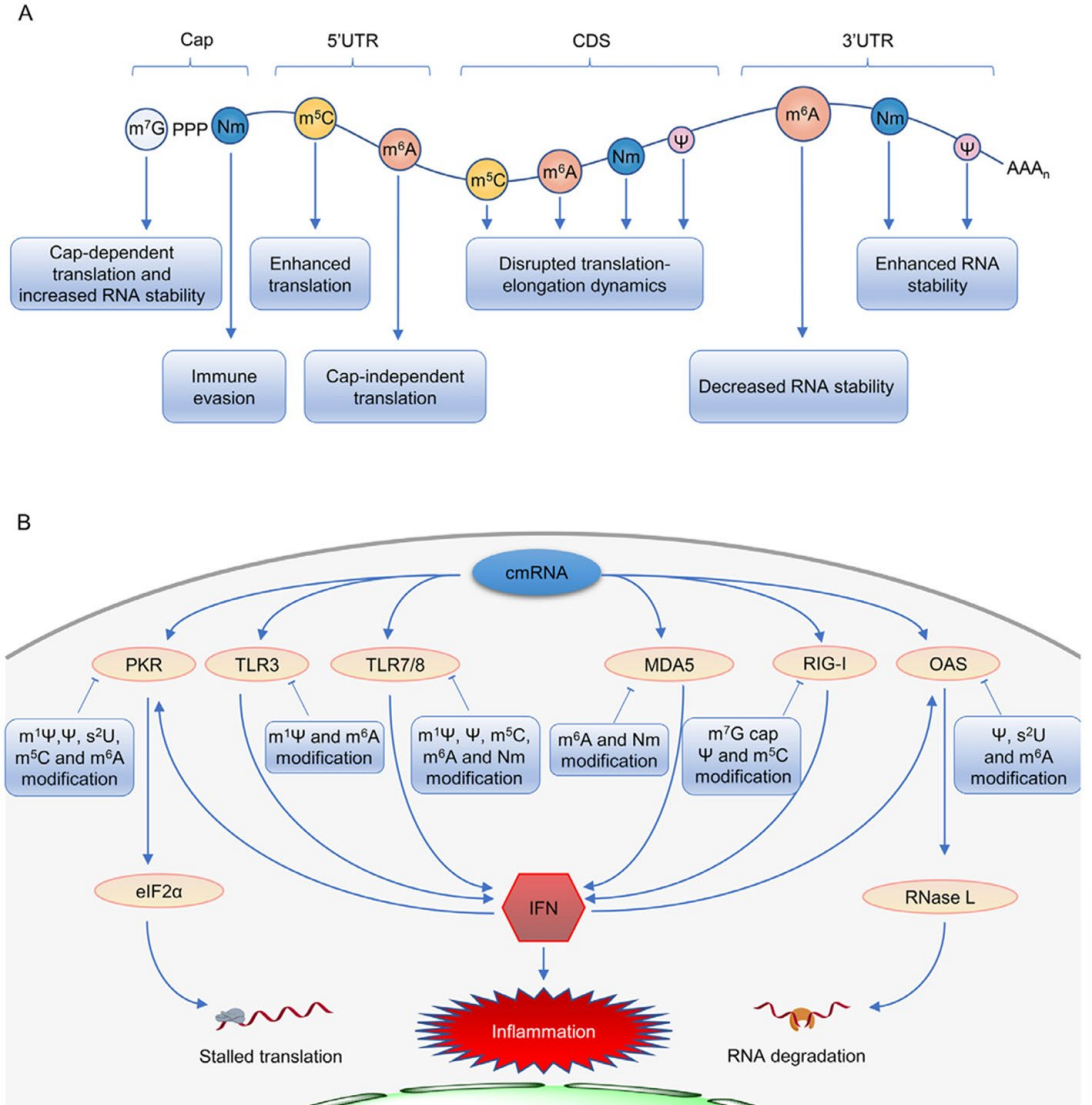
Use of Modified mRNA Therapy in Prevention of Cardiac Remodeling [69]. Copyright 2021, with permission from Elsevier

Recent advances in delivery technologies, purification processes, and nucleoside modifications have addressed many of the limitations historically associated with mRNA-based therapeutics. These developments position mRNA as a promising modality for diseases with limited treatment options, particularly in oncology [65, 66] and cardiology [67, 68]. A major advantage of mRNA over DNA-based approaches is that it does not require nuclear entry, thereby simplifying delivery and reducing integration-related risks. However, unmodified mRNA is immunogenic, a challenge that has been largely mitigated by chemical modifications, allowing more stable and controlled protein expression (Fig. 4).

From an industrial perspective, translation of protein replacement therapies requires not only proof of biological activity but also robust processes for large-scale manufacturing. For instance, scalable in vitro transcription (IVT) platforms, downstream purification methods capable of removing dsRNA contaminants, and formulation technologies compatible with GMP requirements are essential. Translate Bio’s CFTR mRNA therapy illustrates how a lab-scale concept can progress into Phase I clinical testing. It also highlights the need for reproducible, high-yield mRNA production and stability testing that can meet regulatory expectations [70]. Similarly, The VEGF-A modified mRNA program later developed as AZD8601 provides another example of how early mechanistic studies can be translated through biotechnology–pharma collaboration [71].

Compared with DNA-based gene therapies, transient protein expression can be viewed as a limitation. However, this feature may be advantageous in clinical settings that require controllable and repeat dosing. Further industrialization will depend on more detailed pharmacokinetic/pharmacodynamic (PK/PD) characterization, improved stability during storage and transport, and cost-effective production at commercial scale.

### mRNA vaccines for the prevention of infectious disease

Vaccination remains one of the most important public health interventions, but traditional vaccine platforms can be difficult to adapt quickly during emerging outbreaks. mRNA vaccines help address this limitation by allowing rapid antigen design and flexible manufacturing [72, 73]. The COVID-19 pandemic demonstrated that lipid nanoparticle (LNP)-encapsulated mRNA vaccines can be developed, scaled, and distributed globally within unusually short timelines, providing strong evidence of their industrial feasibility.

From an industrial perspective, mRNA vaccines are valuable because they are compatible with cell-free manufacturing, modular process design, and relatively short production cycles. In vitro transcription (IVT) reactions can be rapidly adapted to encode different antigens, while LNP formulation platforms can be standardized for multiple vaccine candidates. This modularity supports multi-product pipelines within the same facility and can improve capital efficiency. However, cold-chain requirements remain a major barrier to global access and continue to drive the need for more stable formulations.

### Genome editing

The emergence of genome editing technologies, particularly CRISPR–Cas9, has further broadened the scope of mRNA therapeutics. Delivery of Cas9 mRNA together with sgRNAs enables transient but efficient genome editing without permanent integration of foreign genetic material [74]. Ex vivo applications, such as hematopoietic stem cell editing for sickle cell disease and CAR-T engineering for oncology, are currently the most clinically advanced, whereas in vivo applications are still under active investigation [75].

Industrializing genome editing therapies presents challenges beyond demonstrating scientific feasibility. Manufacturing must support the consistent GMP production of multiple components, including Cas9 mRNA and sgRNAs, together with validated delivery platforms such as LNPs. Scalable processes also need to maintain reproducible editing efficiency across batches while minimizing off-target activity.

**Fig. 5:**
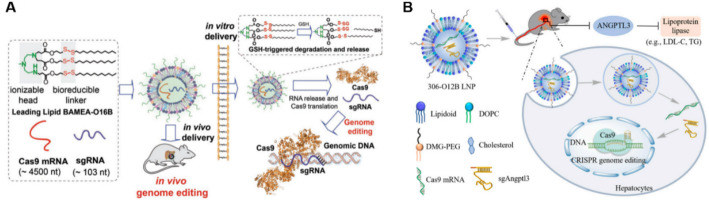
Integrating codelivery and bioreducible chemistry for liver-directed CRISPR/Cas9. **A** Schematic of bioreducible lipid nanoparticles co-encapsulating Cas9 mRNA and single-guide RNA, with a workflow spanning in vitro transfection and systemic administration for in vivo genome editing. Copyright © 2019 Wiley‐VCH Verlag GmbH & Co. KGaA **B** LNP-mediated in vivo editing of Angptl3 in hepatocytes, producing loss of function and reductions in circulating lipid levels. Copyright 2021 National Academy of Sciences

Recent preclinical demonstrations, such as LNP-mediated in vivo editing of PCSK9 [76] and ANGPTL3 [77] in the liver (Fig. 5), illustrate both the therapeutic potential and the need for robust industrial processes to ensure consistency across large patient populations. To translate these findings into approved therapies, the field must integrate advances in nanoparticle engineering with scalable manufacturing and regulatory-compliant quality control systems.

## Properties of lipid nanoparticles (LNPs)

### Size and distribution

For commercial development, particle size and its distribution are regarded as critical quality attributes (CQAs), as they dictate biodistribution, clearance, and ultimately therapeutic efficacy [78]. While preclinical studies suggest optimal ranges between 50–150 nm, industrial production emphasizes not only achieving these targets but also ensuring batch-to-batch reproducibility. Controlled rapid-mixing systems, including microfluidic and impinging-jet approaches, are widely used because size can be tuned by flow rate ratio, total flow rate, lipid composition, and PEG-lipid content (Table 5) [79].

**Table 5:** Engineering comparison of microfluidic scale-out, impinging-jet scale-up, and bulk mixing for LNP manufacturing

Dimension	Microfluidic scale-out	Impinging-jet scale-up	Bulk mixing / ethanol injection
Scale principle	Numbering-up or parallelization of chips or larger channels	Throughput increased by jet velocity, chamber geometry, and flow rate while preserving turbulent micro-mixing	Volume-based scale-up in stirred vessels or semi-batch addition
CPP sensitivity	FRR, TFR, channel geometry, pressure balance, clogging, pH, and ethanol dilution	Jet velocity/Reynolds number, impingement geometry, residence time distribution, pressure, FRR, and TFR	Mixing intensity, addition rate, impeller/vessel geometry, and local ethanol/pH gradients
Reproducibility	High at small scale; scale-out requires chip-to-chip and manifold comparability	High throughput if turbulent regime and mixer geometry are controlled	More vulnerable to mixing heterogeneity and operator/process variability
Automation/closure	Amenable to closed automated lines; disposable chips may be used	Well suited to closed continuous manufacturing and PAT/IPC integration	Conventional equipment is widely available, but real-time control can be harder
Cleaning/validation	Device-specific cleaning or single-use validation; fouling/clogging risk	Mixer and line validation; high-pressure operation; single-use or cleanable flow paths	Established CIP/SIP workflows, but larger hold-up volumes
Cost/COG drivers	Chips, manifolds, pumps, pressure monitoring, and disposables	High-throughput mixers, pressure/control systems, and downstream TFF capacity	Large reactors, utilities, labor, and longer process times
Regulatory/comparability burden	Demonstrate equivalent CQAs across parallel chips/devices	Demonstrate equivalent mixing regime and CQAs after geometry or throughput changes	Justify homogeneity, CQA control, and batch comparability

Regulatory authorities now expect robust in-process controls (IPC) and validated analytical methods, typically dynamic light scattering (DLS) and nanoparticle tracking analysis (NTA), to monitor particle size and polydispersity index (PDI) across GMP production. Failure to maintain a narrow size distribution can affect both pharmacokinetics and patient safety, making particle size a central parameter in regulatory filings. However, DLS and NTA alone are not sufficient to define LNP structure in a CMC dossier. DLS provides an intensity-weighted hydrodynamic diameter and is therefore strongly influenced by larger particles or low-level aggregates. NTA offers number-weighted particle tracking, but its accuracy depends on optical contrast, dilution conditions, and detection thresholds. Importantly, neither method directly captures internal morphology, lamellarity, bleb formation, mRNA spatial distribution, or lipid phase organization. These structural features may affect encapsulation stability, endosomal escape, potency, and batch comparability, yet remain invisible in conventional size and PDI measurements [80, 81].

### Surface charge

Surface charge, commonly reported as zeta potential, affects cellular uptake and toxicity. Ionizable lipids have become important in industrial LNP development because they help balance tolerability and delivery efficiency [82]. They remain nearly neutral at physiological pH, reducing nonspecific interactions in circulation, but become positively charged in acidic endosomes to promote payload release.

For GMP manufacturing, charge consistency should be verified, typically by electrophoretic light scattering. Zeta potential is generally expected to remain within a predefined product-specific range, often close to neutral for systemic LNP formulations. In addition, surface charge can be tuned through lipid composition and the nitrogen-to-phosphate (N/P) ratio. This makes charge modulation not only a formulation variable, but also a process-relevant parameter that should be controlled and validated during scale-up [33].

### Lipid encapsulation and phase behavior

Encapsulation efficiency is a key performance indicator (KPI) for mRNA-LNP therapeutics [83]. In industrial development, values above 90% are often targeted to reduce free RNA degradation and support accurate dosing. Lamellar and non-lamellar lipid phases both contribute to RNA encapsulation and release. From a manufacturing perspective, however, the main challenge is to maintain high encapsulation efficiency consistently across scales, from clinical production to commercial manufacturing.

Lipid phase behavior can also complicate manufacturing. pH-dependent transitions may influence release kinetics, which makes tight control of buffer composition and mixing conditions essential. Regulatory submissions increasingly require a mechanistic understanding of how phase behavior relates to product stability and performance [84, 85]. This understanding should be supported by biophysical assays integrated into the development workflow.

CMC-oriented characterization of mRNA-LNPs should therefore go beyond conventional size and PDI measurements. Cryo-TEM or cryo-EM can directly visualize particle morphology, lamellarity, bleb-like structures, and structural heterogeneity. SAXS and SANS provide ensemble-level information on internal ordering and lipid phase behavior. SEC-MALS or AF4-MALS can help resolve aggregates and particle subpopulations that may be masked in DLS measurements. LC–MS-based assays are needed to quantify lipid composition and impurity profiles, while cell-based potency assays remain essential for linking physicochemical CQAs with biological function [80, 81].

### Stability and storage

Stability remains one of the most visible obstacles in LNP industrialization. mRNA-LNP products are sensitive to RNA hydrolysis, lipid oxidation, aggregation, leakage, and structural damage during freezing or long-term storage. Early deployment of COVID-19 vaccines made this issue clear: different products required different frozen-storage conditions, reflecting differences in formulation composition, cryoprotectant selection, and stability control.

Storage-buffer stability, however, is only part of the problem. After administration, LNPs encounter plasma proteins, lipoproteins, salts, enzymes, and complement components. In this environment, they may acquire a protein corona, exchange lipids with lipoproteins, lose PEG-lipid from the surface, aggregate, partially disassemble, or release mRNA. These changes can alter apparent size, surface properties, uptake route, organ distribution, endosomal escape, and immune activation. Plasma or serum stability should therefore be treated as a translational CQA, not as an optional exploratory test. Incubation in biological fluids followed by SEC-MALS, AF4-MALS, cryo-TEM, LC–MS lipid profiling, free mRNA quantification, complement assays, and potency testing provides a more realistic view of whether an LNP formulation remains intact after physiological exposure [86].

Chemical degradation adds another layer of risk. Many ionizable lipids contain tertiary amines, ester linkages, or unsaturated hydrophobic chains that can undergo oxidation, hydrolysis, or peroxide-mediated degradation. Tertiary amines may form N-oxide species, while lipid-chain oxidation can generate aldehydes and other reactive carbonyl compounds. These degradants can react with nucleophilic sites on mRNA and form covalent mRNA-lipid adducts, reducing RNA integrity and translation efficiency. Stability programs for mRNA-LNPs therefore need to monitor not only size, PDI, encapsulation efficiency, and RNA integrity, but also lipid degradants, N-oxide and aldehyde impurities, mRNA-lipid adducts, and potency after accelerated and long-term storage [87, 88].

Industrial viability also depends on practical stability measures. These include cryopreservation and lyophilization with sugars such as sucrose or trehalose to reduce ice-related damage [89, 90], shelf-life programs aligned with ICH expectations, and cold-chain logistics that can support global distribution without excessive cost or product loss.

The next generation of LNP products is likely to rely on a combination of lyophilization, improved cryoprotectant systems, chemically more stable lipid scaffolds, and biodegradable lipid chemistries. Such approaches could lower distribution costs and improve access in low-resource settings. More importantly, stability development should move beyond empirical storage optimization and toward an integrated control strategy that considers storage stability, physiological stability, lipid degradation chemistry, mRNA-lipid adduct formation, and potency retention [86–88].

### Manufacturing, control strategy, and regulatory lifecycle management of mRNA-LNP products

In real development programs, CMC success often depends on issues that are easy to underestimate. These include lipid raw-material qualification, supplier variability, lot-to-lot propagation of CQAs such as size, free mRNA, and potency, analytical sensitivity for complex nanoparticles, and stability constraints that affect cold-chain cost and access. Addressing these issues requires both improved chemotypes, such as biodegradable linkers, and robust control strategies with defined CPP windows, IPC/PAT tools, and explicit comparability protocols.

Regulatory expectations for lipid-based nanomedicines have become more defined over time. Relevant documents include FDA guidance on liposome drug products [91] and and products containing nanomaterials [92], the EMA reflection paper on intravenous liposomal products developed with reference to an innovator product [93], and ICH Q12 guidance on lifecycle management of post-approval CMC changes [94].

Comparability, scale-up bridging, and post-approval change management. Because mRNA-LNPs are complex nanomedicines, changes in mixing technology, manufacturing site, scale, lipid supplier, or stabilization strategy can shift CQAs. A practical approach is to define a comparability plan early, including acceptance criteria, orthogonal analytical characterization, and, when necessary, nonclinical or clinical bridging. After approval, change management should remain risk-based and supported by process understanding and validated analytical methods.

Moving from laboratory LNP preparation to commercial manufacturing is not a simple increase in volume. Microfluidic devices offer strong control of particle size and PDI at small scale by controlling local mixing, ethanol dilution, and nucleation kinetics. Industrial scale-out, however, requires reliable parallelization, balanced flow distribution, pressure control, clogging prevention, chip-to-chip comparability, and cleaning or single-use validation. Confined impinging-jet mixing is often better suited to high-throughput production because it uses turbulent mixing and can be engineered for larger flow rates. Even so, scale-up requires comparable mixing time, Reynolds number, jet velocity, inlet geometry, and residence time distribution. Changes in mixer geometry can alter ethanol dilution and lipid self-assembly, which may shift particle size, PDI, encapsulation efficiency, internal morphology, and potency. Mixer transfer or scale increase should therefore be treated as a major CMC comparability event rather than a routine process adjustment [79].

Critical quality attributes and linkage to process parameters. For LNPs, relevant CQAs usually include particle size distribution and PDI, encapsulation efficiency and free mRNA content, potency, lipid composition and identity, residual solvents, pH and osmolality, impurity profile, sterility and endotoxin, and stability under intended storage conditions. CPPs that commonly drive these attributes include aqueous pH and ionic strength during mixing, ethanol fraction, lipid:mRNA ratio or N/P ratio, total flow rate and flow rate ratio for microfluidic systems, mixing time or energy for bulk processes, and downstream shear or pressure during filtration.

Industrial translation also requires an end-to-end control strategy that connects formulation choices with manufacturing operations and final product specifications. A typical GMP workflow includes lipid synthesis and qualification, IVT production and purification of mRNA with control of dsRNA and other process impurities, nanoparticle formation by controlled mixing, solvent removal and buffer exchange, usually by tangential flow filtration, sterile filtration and fill-finish, and frozen or lyophilized storage. Each unit operation can affect CQAs and should therefore be justified within a QbD framework.

### Critical translational risks in mRNA-LNP development

Translational failure of LNP products is rarely caused by one variable alone. More often, problems arise when chemical, structural, and process-related changes are not captured by the selected analytical package. A lipid batch may meet a nominal purity specification but still contain low levels of oxidation products, N-oxide species, aldehydes, peroxides, or regioisomeric impurities that affect mRNA stability during storage [87, 88]. Likewise, a change in mixer geometry, flow rate, manufacturing site, or lipid supplier may leave size and PDI apparently unchanged while altering internal morphology, lamellarity, encapsulation state, or biological activity [79–81]. Physiological exposure adds further uncertainty. A formulation that appears stable in buffer may behave differently in plasma or serum because of protein corona formation, lipid exchange with lipoproteins, PEG-lipid desorption, or complement interaction [86]. Bridging studies can therefore fail when comparability depends mainly on DLS size, PDI, and encapsulation efficiency without orthogonal structural, chemical, and potency assays [80, 81].

A stronger CMC strategy should combine mechanism-informed raw-material control, stability-indicating impurity profiling, orthogonal structural characterization, physiological stability testing, validated potency assays, and predefined comparability protocols [79–81, 86–88]. For scale-up and post-approval changes, acceptance criteria should be built around a connected CQA panel rather than a single particle-size or encapsulation metric. This approach reduces the chance that apparently minor chemical or engineering changes will translate into meaningful differences in biodistribution, immunogenicity, or potency.

## Delivery platform companies

**Fig. 6:**
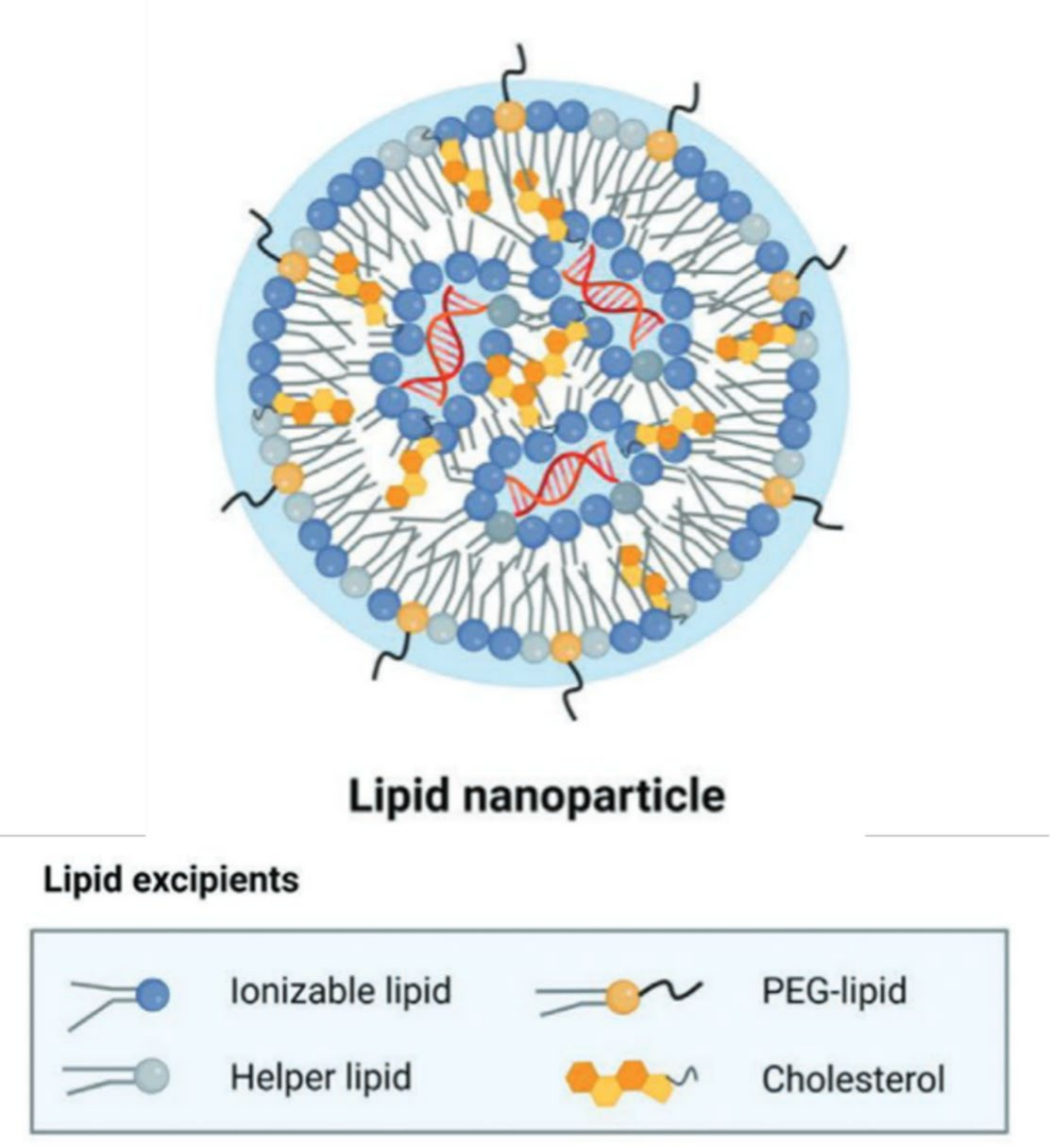
Schematic components of mRNA LNPs. [95] Copyright © 2022 Wiley‐VCH GmbH

The industrialization of RNA therapeutics has progressed in parallel with delivery technologies, and LNPs have become the most widely adopted platform. Their modular architecture, usually based on ionizable lipids, helper lipids, cholesterol, and PEG-lipids, protects RNA cargo and supports controlled intracellular release (Fig. 6). Industrial progress, however, is not achieved by formulation optimization alone. It also requires scalable processes, resilient supply chains, and compliance with increasingly detailed regulatory expectations.

Over the past decade, biotechnology companies have helped define the RNA delivery ecosystem. Some have focused on lipid design, including cholesterol- and non-cholesterol-based lipids for gene therapy and vaccine delivery [96], or modular LNP systems that can be adapted to different nucleic acid payloads [97]. Others have emphasized engineering, especially microfluidic continuous production, which supports reproducible GMP-compliant scale-up from preclinical to clinical manufacturing [57]. Selective organ and tissue targeting has also become a major direction as RNA medicine moves toward more precise delivery [98]. These strategies show how lipid chemistry, formulation flexibility, scalable processing, and targeting specificity all contribute to the movement of LNPs from laboratory systems to clinical products.

The growth of RNA delivery platforms has also changed the CDMO landscape. Companies such as CordenPharma, Evonik, and Samsung Biologics have invested in lipid synthesis and RNA formulation capacity, accelerated by the COVID-19 vaccine rollout. That experience showed that global production of RNA-LNP medicines is feasible, but it also exposed weaknesses in lipid sourcing, quality assurance, and cold-chain logistics. A more sustainable supply chain will require diversified lipid suppliers, integrated production systems, and stronger distribution networks as RNA medicines expand beyond pandemic use.

**Fig. 7:**
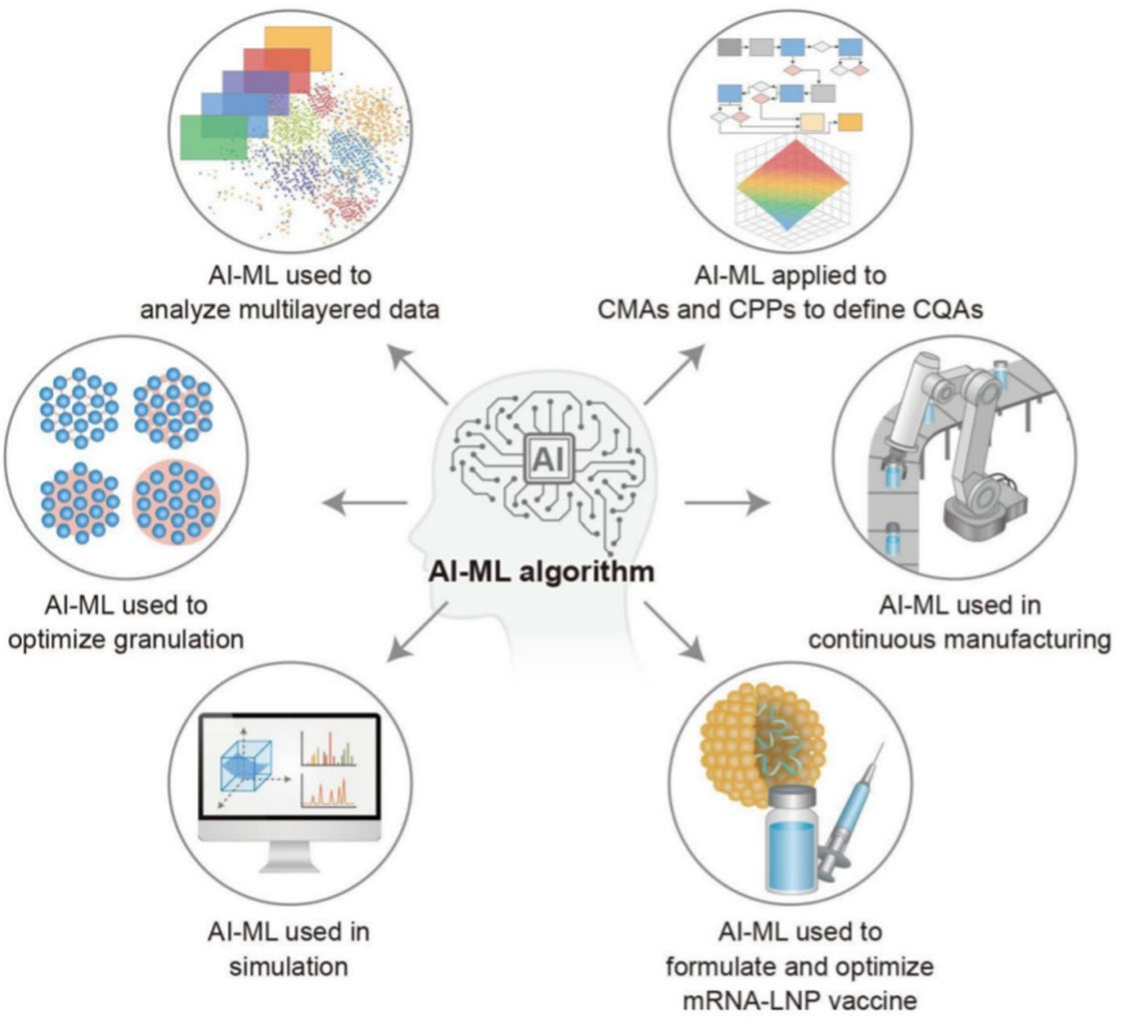
Application of AI-ML tools in the pharmaceutical and biopharmaceutical industries [101]. Copyright 2023, Springer Nature

Artificial intelligence (AI) and machine learning are beginning to influence RNA delivery development [99–101]. Traditional formulation optimization still depends heavily on empirical screening, which is slow and resource intensive. AI-driven workflows can analyze larger experimental datasets, identify structure–activity relationships, and predict formulation outcomes (Fig. 7). For LNPs, these predictions may include how lipid composition affects encapsulation efficiency, size distribution, endosomal escape, and in vivo biodistribution.

The current value of AI/ML for LNP formulation design is nevertheless limited by the datasets used to train the models. Many published datasets are relatively small and differ in mRNA construct, lipid library, mixer, buffer, animal model, and potency assay. As a result, strong model performance may reflect dataset-specific correlations rather than generalizable formulation principles. For AI-driven QbD to become practical, LNP datasets need standardized, machine-readable metadata so that material attributes, CPPs, CQAs, and potency readouts can be compared across laboratories and manufacturing scales [79–81, 102]. Table 6 summarizes a practical minimum metadata scheme.

**Table 6:** Proposed minimum metadata fields for AI/ML-ready and QbD-relevant mRNA-LNP formulation datasets

Metadata domain	Minimum fields	Purpose for QbD/AI interpretation
Lipid chemistry and raw materials	Ionizable/helper/sterol/PEG-lipid structures or identifiers; molar ratios; lipid batch/supplier; purity; impurity/degradant profile; apparent pKa; degradable linker or targeting motif	Connects critical material attributes (CMAs) to CQAs, potency, tolerability, and stability
RNA payload	mRNA length; sequence class; cap; UTR/poly(A) design; nucleotide modification; RNA concentration; dsRNA/process-related impurities; N/P ratio	Avoids confounding formulation performance with payload quality or construct effects
Manufacturing process	Mixer type and geometry; FRR; TFR; ethanol fraction; aqueous pH/ionic strength; temperature; residence time; dilution/quench; TFF/filtration conditions	Links CPPs to particle formation, internal structure, residual solvent, and reproducibility
Product CQAs	Size/PDI; zeta potential; encapsulation efficiency/free RNA; morphology/lamellarity; lipid composition; residual ethanol; osmolality/pH; sterility/endotoxin	Defines comparable outputs for release testing, comparability, and model labels
Stability and biological readouts	Storage/accelerated-stress conditions; serum/plasma incubation; free RNA after exposure; lipid degradants/adducts; potency assay system; dose/route; cell/animal model; time point	Enables models to distinguish storage stability, physiological stability, and biological efficacy

Regulatory frameworks have simultaneously evolved, with lipid-based nanoparticle products increasingly treated as complex nanomedicine or liposomal/nanomaterial-related products that require product-specific CMC justification. This places substantial demands on documentation of chemistry, manufacturing, and controls. Critical quality attributes include particle size distribution and reproducibility, surface charge and ionization behavior, encapsulation efficiency and drug loading, phase behavior and morphology under physiological and storage conditions, impurity profile, release kinetics, potency, and long-term stability. The approval of mRNA vaccines by Moderna and Pfizer-BioNTech has established regulatory benchmarks, and nanomedicine-specific guidance continues to be refined to ensure batch-to-batch consistency and global reproducibility.

Several industrial trends are now clear. Strategic collaborations and licensing models, including BioNTech-Pfizer, Moderna-Lonza, and Acuitas Therapeutics partnerships, have helped consolidate the LNP ecosystem. Developers are also pursuing thermostable and lyophilized formulations to reduce cold-chain constraints, while continuous microfluidic systems are being adopted for closed, automated, and scalable manufacturing. Sustainability is becoming more visible as well, with attention to lipid synthesis, greener production methods, and cost efficiency. Beyond infectious diseases, industrial development is expected to expand into oncology, rare genetic disorders, and regenerative medicine, where delivery remains a decisive factor.

## Conclusion

RNA therapeutics have moved from a long-standing concept to a clinically validated modality, and LNPs have played a central role in this transition. The success of mRNA vaccines during the COVID-19 pandemic provided regulatory precedent, demonstrated the feasibility of global manufacturing, and highlighted the importance of supply-chain resilience. At the same time, formulation complexity, physiological stability, lipid degradation chemistry, cold-chain requirements, and long-term tolerability continue to shape research priorities and industrial investment.

The next stage of RNA delivery development will depend on progress in several connected areas. Manufacturing platforms must become more scalable and reproducible, whether through microfluidic scale-out, impinging-jet scale-up, or closed continuous processing. Lipid chemistry needs to continue improving safety, biodegradability, and storage stability. Regulatory strategies also need to keep pace with the complexity of LNP products by incorporating orthogonal comparability, physiological-stability assessment, and stability-indicating impurity profiling.

As RNA therapeutics move beyond vaccines into oncology, rare genetic disorders, genome editing, and regenerative medicine, delivery will remain a major determinant of success. Closer coordination among academic groups, biotechnology companies, CDMOs, analytical-technology providers, and regulators will be needed to connect discovery-stage innovation with manufacturable products. With better lipid design, more standardized datasets, and end-to-end CMC control, mRNA-LNP products are likely to remain a central platform for precision and personalized medicine.

## Data Availability

Not applicable.
